# Spontaneous hemoperitoneum caused by meckel’s diverticulum in an elder patient

**DOI:** 10.11604/pamj.2016.24.314.10384

**Published:** 2016-08-17

**Authors:** Adriá Rosat, Eduardo Pérez, Hanna Hernández Oaknin, Javier Mendiz, Guillermo Hernández, Manuel Barrera

**Affiliations:** 1 Department of General Surgery, Hospital Universitario Nuestra Señora de Candelaria, Ctra Del Rosario 145, 38010 Sta. Cruz de Tenerife, Spain; 2 Transplantation Surgery Unit and General Surgery Service, Hospital Universitario Nuestra Señora de Candelaria, Ctra. Del Rosario 145, 38010 Sta Cruz de Tenerife, Spain

**Keywords:** Meckel´s diverticulum, spontaneous hemoperitoneum, old patient

## Abstract

Symptomatic Meckel’s Diverticulum is a rare entity in old patients. The most common complications are gastrointestinal bleeding, intestinal obstruction, acute inflammation, and perforation. Usually those complications occur on the first two decades of life and mostly before the fifth decade. We report an extremely unusual debut of Meckel’s Diverticulum, presented as massive spontaneous hemoperitoneum in an 82-year-old man without gastrointestinal bleeding. A literature review of atraumatic hemoperitoneum as presentation of Meckel’s diverticulum was made.

## Introduction

Meckel’s diverticulum, the result of incomplete obliteration of the omphalomesenteric (vitelline) duct, is the most common congenital abnormality of the small bowel [1]. Ninety percent occur within the terminal 90 cm of ileum [2]. Meckel’s diverticulum is a “true” diverticulum, therefore; it has all layers of the ileal wall. Recognizing a 2% incidence of Meckel’s diverticulum in the general population, the surgeon can use life-table analysis to calculate the risk of the development of future complications from Meckel’s diverticulum. According to current literature the rate of complication progressively descend with age from 3% at 10 years to 0% at 75 years [3]. The most common complications include gastrointestinal bleeding, intestinal obstruction due to internal herniation or intussusceptions, and perforation [3, 4]. Less frequently enconuntered are sinus tract formation, internal fistulas and tumors. Intestinal volvulus and torsion can occur around the mesodiverticulat band that is associated with approximately 10% of cases [4]. We report an extremely rare complication of perforated Meckel’s diverticulum, which presented as spontaneous hemoperitoneum.

## Patient and observation

An 82-year-old male was admitted in our outside hospital’s emergency room due to abdominal pain and faint. He had a previous history of auricular fibrillation in treatment with a dicumarinic anticoagulant and a previous episode of abdominal pain which was diagnosed as spontaneous mesenchimal haematoma and treated non operatively.

**Figure 1:**
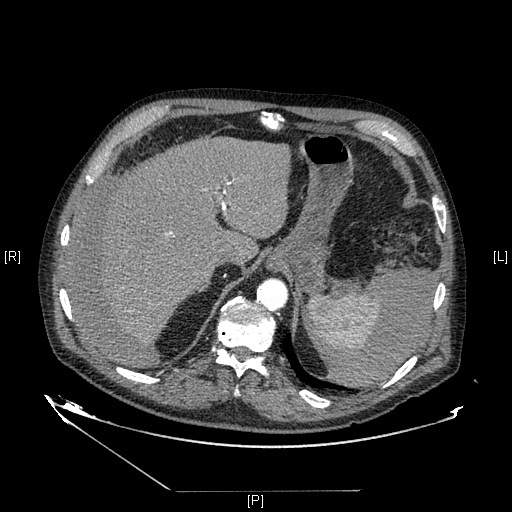
Perihepatic and perisplenic hemoperitoneum

**Figure 2:**
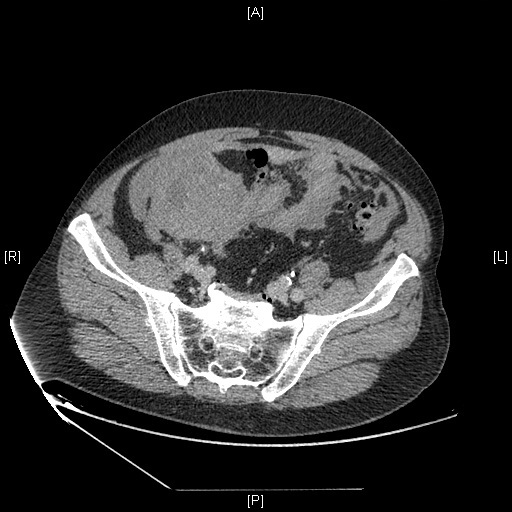
Expansive mesenteric hematoma

He denied any kind of trauma, and had no hematemesis or melena. At physical examination the patient was stable and suffered from intense pain at the right lower abdomen with no defense. Blood tests showed a haemoglobin level of 10 g/dl. Coagulation was altered as expected. CT scan showed massive hemoperitoneum (Figure 1) and the already known mesenteric hematoma (Figure 2). Assuming the diagnosis of expansive mesenteric hematoma plus ongoing bleeding a laparotomy was performed.

**Figure 3:**
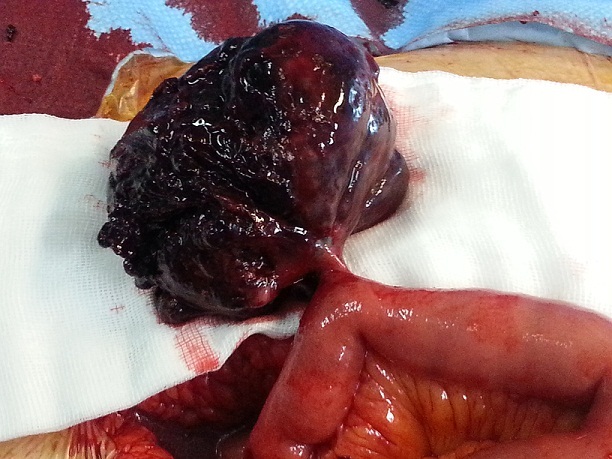
The hemorrhagic diverticulum with torsionated pedicle

During first exploration, 3 liters of fresh and old blood clots were found. At 90cm from the ileocecal valve a torsionated and perforated meckel diverticulum with intradiverticular bleeding was found (Figure 3) and a diverticular resection was performed. The postoperatory was uneventful and the patient was discharged home at the 9th postoperative day.

## Discussion

To our knowledge, there have been only few reports of a hemoperitoneum caused by a Meckel’s diverticulum without previous abdominal trauma in the world literature [5–16]. In most of the cases (66%), hemoperitoneum is discovered as a result of peptic ulceration and perforation of a Meckel’s diverticulum [5–12].

In four cases the Meckel’s divercitulum remained nonperforated and the bleeding appeared to be a consequence of local inflammation caused by Meckel’s diverticulitis resulting in a disruption of the diverticulum’s vascular supply [13–16]. Involved mechanism in these cases could be the tearing of mesodiverticular bands, frequently detached and not seen during the surgical exploration, which often contain a vascular component. Injury to these bands has been associated with intraperitoneal bleeding in both traumatic and atraumatic settings.

This case has 2 unique features. First, it is unusual for Meckel´s diverticulum to cause symptoms in such an old patient. And second, the bleeding was only intraperitoneal, he had not intraluminal bleeding. Perhaps the most likely explanation is the possibility that the pedunculated diverticulum had undergone axial torsion with resultant venous engorgement and ischaemia followed by spontaneous intraabdominal bleeding, avoiding this way any intraluminal bleeding sign. However, this is not supported by the absence of any fibrous or fibrinous attachment to provide a pivot point which may encourage axial torsion. The absence of an attachment at operation to the tip of the diverticulum itself need not necessarily mean it was not present, for this may have become necrotic during the previous episode or detached during the surgical exploration. Torsion of an unattached pedunculated appendage with a heavier bulbous end such as this may still occur akin to the mechanism of torted ovarian cyst, clapper-bell testis or appendix epiploicae. The surgical exploration itself could have caused reversal of torsion. Torsion, with or without ileal volvulus, is a recognized complication of Meckel’s diverticulum.

## Conclusion

The preoperative diagnosis of a Meckel’s diverticulum is notoriously difficult in the adult patient, with the diagnosis being secured in the operating room in most of the cases. A complicated Meckel’s diverticulum, although rare, should be recognized among causes of hemoperitoneum, and should be considered after the more common causes of a hemoperitoneum have been ruled out.
